# Safe stool disposal and associated factors among mothers of children aged under-two years in Gambia: Evidence from Gambia Demographic Health Survey 2019/20

**DOI:** 10.1371/journal.pone.0284986

**Published:** 2023-05-01

**Authors:** Menen Tsegaw, Bezawit Mulat, Kegnie Shitu

**Affiliations:** 1 Department of Public Health, College of Medicine and Health Sciences, Ambo University, Ambo, Ethiopia; 2 Department of Human Physiology, School of Medicine, College of Medicine and Health Sciences, University of Gondar, Gondar, Ethiopia; 3 Department of Health Education and Behavioral Sciences, Institute of Public Health, College of Medicine and Health Sciences, University of Gondar, Gondar, Ethiopia; Cranfield University, UNITED KINGDOM

## Abstract

**Background:**

Appropriate disposal of child stool is vital in preventing the spread of faecal-oral diseases. According to WHO/ UNICEF Joint Monitoring Program, Safe child stool disposal includes disposing a stool in a Flush or pour-flush toilet/latrine (to a piped sewer system, septic tank, pit latrine), Ventilated improved pit (VIP) latrine or a Pit latrine with slab.

**Objective:**

The study aimed to assess safe child stool disposal practice and associated factors among mothers with children aged under-two years in Gambia.

**Methods:**

This study was based on a large community-based cross-sectional survey, conducted from 21 November 2019 to 30 March 2020 in Gambia. The survey employed a two-staged stratified cluster sampling technique to recruit study participants. Descriptive statistics and logistic regression models were used to summarize descriptive data and identify factors associated with safe waste disposal, respectively. A p-value of less than 0.05 and 95% confidence interval were used to determine statistical significance.

**Results:**

The prevalence of safe stool disposal among mothers with children aged under-two years were 56.3% (95% CI: 54.6%, 58.1%). Mothers aged 25–34 (AOR = 0.78 (95%CI: 0.62, 0.98)), the highest wealth quintile (AOR = 0.43 (95%CI: 0.33, 0.56)), being exposed to media (AOR = 1.37 (95%CI: 1.07, 1.76)), increasing age of children (AOR = 1.06 (1.05, 1.07)), Being employed (AOR = 1.31 (1.11, 1.55)) and Geographic region were significantly associated with safe child disposal practice.

**Conclusion:**

The prevalence of safe child stool disposal was low in Gambia. Age of the mother, age of the child, region, wealth index, media exposure and occupational status of the mother were significantly associated with safe child stool disposal. Public health intervention strategies designed to promote safe child stools disposal need to conduct thorough community assessments to identify community-specific facilitators, needs and barriers. Additionally, public health experts and policy makers should take into consideration the geographical and wealth inequalities when designing programs aimed to improve safe child stool disposal practice.

## Introduction

According to WHO/ UNICEF Joint Monitoring Program, Safe child stool disposal is defined as disposing stools in one of the following options: Flush or pour-flush toilet/latrine (to a piped sewer system, septic tank, pit latrine), Ventilated improved pit (VIP) latrine or a Pit latrine [1, 2]. Appropriate disposal of child stool is vital in preventing the spread of faecal-oral diseases [3–5]. Sustainable development Goal (SDG) 6 aims to “achieve access to adequate and equitable sanitation and hygiene for all and end open defecation, paying special attention to the needs of women and girls and those in vulnerable by 2030 [6]. Achieving the widespread scope of SDG by 2030 needs quadrupling current rates of advance in securely overseen drinking water administrations, sanitation administrations and basic hygiene services especially in least developing countries [7]. Elimination of open defecation is one of the core priority solutions to reduce global disparities in Water, Sanitation, and Hygiene (WASH) [8].

Different studies revealed that unsafe child stool disposal was one of the major factors that causes enteric infections including Diarrheal diseases [3, 9–13]. Diarrhea is one of the most common causes of under-five mortality rate [14–16]. There is a common misunderstanding that child faeces are less infectious than adult [17, 18]. Besides the Water and Sanitation Project implemented by the Department of Water Resources, to introduce hygienic means of excreta disposal in the entire country, there has not been much-coordinated policy response to basic sanitation issues in Gambia [19]. Different studies have been done on safe child stool disposal and associated factors among children but there is no study done in Gambia. Among the studies that investigated child feces disposal, most found that the maternal age, higher maternal education, highest household wealth index, availability of toilet facility, and improved drinking water were significantly and positively associated safe child stool disposal [20–22]. According to the Gambia Demographic Health Survey (GDHS) 2019/20, safe child stool disposal among children under the age of 2 years has been decreased from 82% in 2013 to 56% in 2019–20. Although the magnitude of safe child stool disposal is known the possible factors affecting safe child stool disposal have not yet been well studied. Therefore, the current study is aimed to assess factors that are significantly associated with safe child stool disposal practice in Gambia using the GDHS 2019–20.

## Methods

### Study design and setting

This study was based on a large community-based survey, Gambia Demographic Health Survey (GDHS), conducted from 21 November 2019 to 30 March 2020 in Gambia. Gambia is located on the West African coast. It is bordered on the North, South and East by the Republic of Senegal and on the West by the Atlantic Ocean. The survey employed a stratified two-stage cluster sampling. In the first stage, Enumeration Areas (EAs) were selected with a probability proportional to their size within each sampling stratum. In the second stage, the households were systematically sampled. A total of 3,011, weighted sample of mothers who have child/children under the age of two years were included in the study.

### Variables of the study

#### Dependent variable

Safe child stool disposal was the outcome variable of this study which was obtained after categorizing child stool disposal in to two as safe **(**Flush or pour-flush toilet/latrine to (piped sewer system, septic tank, pit latrine), Ventilated improved pit (VIP) latrine and Pit latrine with slab) and unsafe **(**pit latrines without a slab or platform, hanging latrines and bucket latrines).

#### Independent variables

Age of the mother, religion, region, educational level, occupation, residence, wealth index, media exposure (yes, no), age of the child, toilet facility (improved, unimproved), sex of the child, birth order, visited a health facility in the last 12 months (yes, no), history of diarrhea in the last 2 weeks preceding the survey (yes, no), source of drinking water (improved, unimproved) and the number of living children were included. Those variables were identified after reviewing different literature [5, 17, 20, 21, 23].

#### Data analysis

The mother’s individual sample weightings were used in the estimation to provide nationally representative results. Descriptive studies like frequency count and proportion for categorical data were used to summarize descriptive data. Bivariable logistic regression was used to select candidate variables for multivariable logistic regression. In the Bivariable logistic regression, a p-value<0.2 was used as a cut of point to select variables for the multi-variable analysis entry. Multivariable logistic regression was used to identify independent predictors of safe child stool disposal in Gambia. A 95% confidence interval (CI) and p-value<0.05 were used to determine the statistical significance. Stata version 14.0 was used for statistical analysis.

## Results

The mean age of the mothers was 28.35 with a standard deviation of 6.65 and range of 15–49 years. Regarding religion, 2,952 (98.0%) of mothers were Muslims. About 1,946 (64.6%) of the mothers were from urban, 1202 (39.9%) were from Brikama region, 1,337 (44.4%) of mothers have no education, 1177 (39.1) of mothers were employed and 1314 (43.6%) of mothers were from the poor families (Table 1).

**Table 1:** Socio-demographic characteristics by waste disposal status among mothers/caregivers of children with the age < 2 years in Gambia, 2019/20 (n = 3011).

Variables	Child waste disposal practice				Total	
	Unsafe		Safe			
	Frequency	Percent	frequency	Percent	Frequency	Percent
Age of the mother						
15–24	367	43.8	471	56.2	838	27.8
25–34	700	44.5	874	55.5	1574	52.3
>34	246	41.1	353	58.9	599	19.9
Region						
Banjul	17	66.9	8	33.1	26	0.9
Kaniking	348	68.7	159	31.3	507	16.9
Brikama	559	46.5	643	53.5	1202	39.9
Mansakonko	23	18.1	106	81.9	129	4.3
Kerewan	153	41.2	219	58.8	372	12.3
Kuntaur	78	41.1	111	58.9	189	6.3
Janjanbureh	40	21.0	152	79.0	193	6.4
asse	95	24.1	299	75.9	394	13.0
Mothers educational level						
No education	514	38.5	822	61.5	1337	44.4
Primary	225	39.3	348	60.7	574	19.1
Secondary	502	51.4	474	48.6	976	32.4
Higher	72	57.5	53	42.5	125	4.1
Occupation						
Employed	612	52.0	565	48.0	1177	39.1
Unemployed	702	38.3	1132	61.7	1834	60.9
Religion						
Islam	1284	43.5	1668	56.5	2952	98.0
Christian	30	49.6	30	50.4	60	2.0
Residence						
Urban	996	51.2	949	48.8	1946	64.6
Rural	317	29.8	748	70.2	1066	35.4
Wealth index						
Poor	427	32.3	887	52.2	1314	43.6
Middle	199	15.1	430	25.3	629	20.9
Rich	688	52.3	380	22.5	1068	35.5
Media exposure						
No	91	6.9	161	9.5	252	9.0
Yes	1223	93.1	1536	90.5	2759	91.0
Age of the child						
0–5 months	502	56.1	394	43.9	897	29.7
6 to 11 months	348	47.5	384	52.5	732	24.3
12 to 17 months	289	36.7	499	63.3	787	26.2
18 to 23 months	175	29.34	421	70.66	596	19.8
Sex of the child						
Male	663	42.6	893	57.4	1556	51.7
Female	651	44.7	804	55.3	1456	48.3
Toilet facility						
Unimproved	388	33.4	773	66.6	1161	38.5
Improved	926	50.0	925	50.0	1851	61.5
Source of drinking water						
Unimproved	136	40.58	199	59.42	334	11.10
Improved	1178	44.01	1499	55.99	2677	88.90
History of Diarrhea						
No	988	44.7	1223	55.3	2211	73.4
Yes	326	40.7	474	59.3	801	26.6
Birth order						
First	307	49.1	318	50.9	625	20.8
Second	260	47.2	291	52.8	552	18.3
Third	241	46.2	281	53.8	523	17.4
Fourth and above	505	38.5	807	61.5	1312	43.5
Visited health facility						
No	115	4.1	128	4.9	2768	9.0
Yes	1198	43.3	1569	56.7	2768	91.9
Number of living children						
1 child	318	48.0	344	52.0	662	22.0
2 children	287	49.1	297	50.9	584	19.4
3 children	249	45.8	295	54.2	544	18.0
4 and above	460	37.7	762	62.3	1222	40.6

### Prevalence of safe children’s stools disposal

The prevalence of safe stool disposal among mothers whose child aged under two years was 56.3% (95%CI: 54.5, 58.1%) (Table 2).

**Table 2:** The percentage of safe stool disposal among mothers with children aged under-two years using GDHS 2019/20 (n = 3011).

variables	Weighted frequency	Weighted percent (95% CI)
Type of toilet facility		
Used toilet/latrine	1697	56.3(54.5, 58.1)
Put/rinsed into drain/ditch	190	6.3 (5.4,7.2)
Throw into garbage	1097	36.4 (34.7,38.1)
Left in the open/not disposed of	22	0.8 (0.4,1.1)
Other	5	0.2 (0.0,0.4)
Child waste disposal		
Safe	1697	56.3 (54.5, 58.1)
Unsafe	1314	43.7 (41.8, 45.4)

### Factors associated with safe children’s stool disposal

In the Bivariable logistic regression age of the mother, religion, region, educational level, occupation, residence, wealth index, media exposure, age of the child, toilet facility, birth order, visited health facility in the last 12 months, and the number of living children, sex of the child, diarrhea history in the last 12months and source of drinking water were independently fitted to the outcome variable. However, only age of the mother, religion, region, educational level, occupation, residence, wealth index, media exposure, age of the child, toilet facility, birth order, visited health facility in the last 12 months, and the number of living children exhibited an association at p-value less than 0.2 and passed to be entered in to the multivariable logistic regression. In the multivariable logistic regression, age of the mothers, occupational status of the mother, household wealth index, region, age of the child and media exposure were significantly associated with safe child stool disposal at p-value of less than 0.05 (Table 3).

**Table 3:** Logistic regression for factors associated with safe stool disposal among mothers with children aged under-two years using GDHS, 2019/20 (n = 3011).

Variables	COR(95%CI)	P-value	AOR(95%CI)	p-value
Age of the mother				
15–24	1		1	
**25–34**	**0.78 (0.79,1.09)**	**0.42**	**0.78 (0.62, 0.98)** *	**0.033**
>34	1.16 (0.94,1.42)	0.17	0.75 (0.53, 1.04)	0.088
Region				
Banjul	1		1	
Kaniking	0.93 (0.61,1.41)	0.73	1.04 (0.67, 1.62)	0.869
**Brikama**	**2.44 (1.66,3.570**	**<0.001**	**2.33 (1.55, 3.50)** *	**<0.001**
**Mansakonko**	**9.10 (5.60,14.26)**	**<0.001**	**5.98 (3.60, 9.94)** *	**<0.001**
**Kerewan**	**2.86 (1.93,4.22)**	**<0.001**	**1.90 (1.22, 2.97)** *	**0.005**
**Kuntaur**	**2.70 (1.83,3.98)**	**<0.001**	**1.64 (1.03, 2.61)** *	**0.037**
**Janjanbureh**	**6.60 (4.36,10.01)**	**<0.001**	**4.48 (2.77, 7.25)** *	**<0.001**
**Basse**	**6.75 (4.58,9.94)**	**<0.001**	**5.01 (3.23, 7.78)** *	**<0.001**
Mothers educational level				
No education	1		1	
Primary	1.04 (0.86, 1.31)	0.71	1.09 (0.89, 1.37)	0.389
Secondary	0.61 (0.52, 0.72)	<0.001	0.97 (0.79, 1.18)	0.747
Higher	0.44 (0.31, 0.61)	<0.001	1.06 (0.65, 1.75)	0.809
Occupation				
Unemployed	1		1	
**Employed**	**1.60 (1.38, 1.85)**	**<0.001**	**1.31 (1.11,1.55)** *	**0.001**
Religion				
Islam	1		1	
Christian	0.63 (0.34, 1.18)	0.15	1.09 (0.54, 2.20)	0.801
Residence				
Urban	1			
Rural	2.27 (1.96,2.62)	<0.001	1.16 (0.90, 1.48)	0.249
Wealth index				
Poor	1		1	
Middle	1.00 (0.83, 1.22)	0.975	1.16(0.92, 1.48)	0.208
**Rich**	**0.29 (0.24, 0.34)**	**<0.001**	**0.43(0.33, 0.56)** *	**<0.001**
Media exposure				
No	1			
**Yes**	**0.87 (0.69, 1.08)**	**0.223**	**1.37 (1.07, 1.76)** *	**0.014**
Age of the child	1.09 (1.06, 1.13)	0.001	1.06 (1.05, 1.07)	<0.001
Sex of the child				
Male	1			
Female	0.97 (0.84, 1.12)	0.69	-	
Toilet facility				
Unimproved	1			
improved	0.61 (0.53,0.70)	<0.001	1.14 (0.95, 1.36)	0.150
Source of drinking water				
Unimproved	1			
improved	0.98 (0.78,1.22)	0.84	-	
Diarrhea in the last two weeks				
No	1			
Yes	1.09 (0.93, 1.28)	0.27	-	
Birth order				
First	1			
Second	1.08 (0.86,1.36)	0.49	1.06 (0.81, 1.38)	0.674
Third	1.08 (0.86,1.36)	0.49	1.07 (0.78, 1.47)	0.680
Fourth and above	1.48 (1.23,1.78)	<0.001	1.39 (0.93, 2.10)	0.106
Visited health facility				
No	1			
Yes	1.25 (0.97,1.60)	0.08	1.08 (0.81, 1.42)	0.600
Number of children born	1.09 (1.06, 1.13)	<0.001	1.01 (0.93, 1.11)	0.841

Note: (-): Not applicable for AOR, (*): statistically significant at p-value<0.05

The odds of safe stool disposal was 22% (AOR = 0.78 (95%CI: 0.62, 0.98)) lower among mothers aged 25–34 as compared with mothers aged 15–24 year. Region of mothers were significantly associated with safe stool disposal. The odds of safe stool disposal were 56% (AOR = 0.43, 95%CI: 0.44, 0.88) lower among rich households as compared to those with poor wealth quantiles. The odds of safe stool disposal was 1.31 (AOR = 1.31 (95%CI: 1.11, 1.55)) times higher among employed women as compared with unemployed mothers.Age of the child was significantly associated with safe stool disposal. As the age of the child increases from 0–2 year the odds of safe stool disposal increases by 6% (AOR = 1.06 (95%CI: 1.05, 1.07).

Media exposure was significantly associated with safe stool disposal. The odds of safe stool disposal were 1.37 (AOR = 1.37 (95%CI: 1.07, 1.76)) times higher among mothers who were exposed to at least one form of media as compared with their counterparts (Table 3).

## Discussions

The study aimed to assess safe child stool disposal and associated factors among mothers having a child under the age of two years. The prevalence of safe stool disposal among mothers whose child was aged below two years of age was 56.3% (95%CI: 54.5%, 58.1%)). The finding of the study is higher than studies done in Eswatini, Ethiopia, Bangladesh, India, and Orissa [3, 17, 22, 24, 25]. On the other hands, the finding of the current study is lower than the study conducted in Malawi, Cambodia and one study which included many countries across Sub-Saharan Africa [21, 26, 27]. The possible reason for the discrepancies might be differences in sample size, sampling technique, method of analysis and differences in population characteristics. The study conducted in Sub-Saharan Africa used large sample size and it incorporates individual and contextual factors of different countries that might affect safe stool disposal practice. contextual variations and commonalities might affect safe disposal of children’s faeces [21, 28].

Region of the mother is associated with safe stool disposal. Mothers from Brikama, mansakonko, kerewan, kuntaur, janjanbureh, Basse were more likely to disposed stool safely as compared with those from Banjul region of Gambia. The possible reason might be different regions of a country may have different access to health care service, infrastructure and different level of health literacy.

Safe stool disposal was significantly associated with the age of the mother. Women in the age group of 25–34 years were less likely to dispose child’s stool safely as compared with women in the age group of f 15–24 years. The finding is consistent with the studies done in Nigeria [29]. This finding is inconsistent with studies done in Sub-Saharan Africa [21]. The possible reason for the discrepancy might be socio-cultural and socio-demographic differences across countries. The other possible reason might be time variation in which our study used only 2019/20 GDHS data but the study in sub-Saharan Africa used DHS data 2015–2018 in 15 sub-Saharan countries. This means, due to time variation there might be different initiatives, policies, strategies and efforts made to improve stool disposal practice among mothers with children aged under-two years.

Safe stool disposal was associated with the age of children. The odds of safe stool disposal increases with increasing child age. This is finding is consistent with the studies done in Malawi, Ethiopia, Eswatini and Ghana [17, 20, 26, 30]. The possible reason might be that as children get older especially starting from 6 months as they start complementary feeding their stool becomes offensive which increases the likelihood of safe stool disposal in older children as compared to younger children less than 6 month whose stool is not offensive making it favorable to defecate on clothes/diapers. The other possible reason might be that some women perceive that faeces of infants especially less than six months are not that much larger and offensive as compared with those who start complementary feeding, which may in turn increases the possibility of disposing the faeces of those older children safely.

Those from households of highest wealth quantiles were less likely to dispose of stool as compared with those from the poor wealth quantiles. Although many studies have revealed that, the highest wealth quantile increases the likelihood of safe waste disposal [5, 18, 22, 23]. The findings of our study are in contrary to those findings. Even though having the highest wealth is important to have easy access to water, sanitation, and hygiene services, it is not a guarantee for safe stool disposal. The highest wealth quantile is necessary but not sufficient to dispose of stools safely. Other factors like knowledge and perception might affect safe stool disposal behavior. That means household income was not associated with safe stool disposal in Gambia. Having high or low wealth quantile is not a precondition to dispose stool safely in Gambia. this implies that during the design of health intervention programs there is a need to consider other factors which may affect stool disposal behavior like knowledge about the importance of safe stool disposal, attitude towards stool disposal and other behavioral as well as socio-cultural issues.

The occupational status of the mothers was significantly associated with safe stool disposal. Mothers who were employed were more likely to dispose of stool safely as compared with unemployed mothers. The finding is inconsistent with a cross-sectional study done in Tigray region of Ethiopia [31]. The possible reason for the discrepancy might be the smaller sample size included in Tigray and the large representative sample used in our study. The other possible reason might be employed mothers have their source of income and they can hire caregivers as well as they can easily access sanitary materials. Therefore, employed mothers would be more likely to dispose of stool safely than mothers who are not employed.

Media exposure was associated with safe child stool disposal. Mothers who were exposed to a media at least once a week were more likely to dispose of stool safely as compared with those who had not exposed. The finding of the study is consistent with what has been reported by a study in in sub-Saharan Africa [21]. Mothers who were exposed to media might get important health information about child waste disposal and its impact on the health of the child as well as the community as a whole, so they might have good knowledge about safe child waste disposal and develop a positive attitude towards the importance of safe child disposal behavior than those mothers who do not exposed. Media has a great impact on behavior change by providing health information to shape the attitude of the community and to promote healthy behavior. Especially Audio-visual education has a great impact on increasing safe child stool practice.

### Strength

Utilization of large sample size and nationally representativeness of DHS data helps to generalize to the population of Gambia. The application of sample weighting to overcome non-proportional sample allocation during the survey. The current study used a large sample size that can help to increase the statistical power and validity of the study.

### Limitations

The main limitation of the study is that since it is a cross-sectional study the temporal relationship between the outcome and independent variables could not be established. The DHS data lacks some variables like knowledge about child stool disposal that may affect their disposal practice. Since the data was collected by using self-reports, there might be a possibility of introducing social desirability bias and recall bias.

## Conclusion

The prevalence of safe child stool disposal was low in Gambia. This might result in diarrheal diseases continued to be an important public health problem, with high morbidity and mortality among children. Safe child stool disposal has a great role in achieving open defecation free communities and reduction of diarrheal diseases. Age of the mother, age of the child, region, media exposure and occupational status of the mother were significantly associated with safe child stool disposal. Household income was not associated with safe stool disposal in Gambia. Public health intervention strategies designed to promote safe child stools disposal need to conduct thorough community assessments to identify community-specific facilitators, needs and barriers. Additionally, public health experts and policy makers should take into consideration the identified factors while designing programs aimed to improve safe child stool disposal practice

## List of abbreviations

COR: (Crude Odds Ratio)
AOR: (Adjusted Odds Ratio)
CI: Confidence interval
GDHS: Gambia Demographic Health Survey
