# Supplementary material for: Safe stool disposal and associated factors among mothers of children aged under-two years in Gambia: Evidence from Gambia Demographic Health Survey 2019/20
Source: PLoS One. 2023 May 1;18(5):e0284986. doi: 10.1371/journal.pone.0284986 (PMC10150983; doi:10.1371/journal.pone.0284986)
Supplement: Attachment — Submitted filename: PONE-D-22-33012_review comments.pdf [file pone.0284986.s001.pdf]

PLOS ONE

Safe stool disposal and associated factors among mothers of children under-two age in Gambia: Evidence from Gambia Demographic Health Survey 2019/20

--Manuscript Draft--

**Manuscript Number:**	PONE-D-22-33012
**Article Type:**	Research Article
**Full Title:**	Safe stool disposal and associated factors among mothers of children under-two age in Gambia: Evidence from Gambia Demographic Health Survey 2019/20
**Short Title:**	Safe stool disposal and associated factors among mothers of children under-two age
**Corresponding Author:**	Menen Tsegaw<br>Ambo University<br>Ambo, Oromia ETHIOPIA
**Keywords:**	safe stool disposal; Gambia
**Abstract:**	Background<br><br>Appropriate disposal of child stool is vital in preventing the spread of faceo-oral diseases. According to WHO/ UNICEF Joint Monitoring Program, Safe child stool disposal includes disposing a stool in to Flush or pour-flush toilet/latrine to (piped sewer system, septic tank, pit latrine), Ventilated improved pit (VIP) latrine and Pit latrine with slab.<br><br>Objective<br><br>The study aimed to assess safe child stool disposal practice and associated factors among mothers having under two age's children in Gambia<br><br>Methods<br><br>This study was based on a large community-based cross-sectional survey, conducted from 21 November 2019 to 30 March 2020 in Gambia. The survey employed a two-staged stratified cluster sampling technique to recruit study participants. Descriptive statistics and logistic regression models were used to summarize descriptive data and identify factors associated with safe waste disposal, respectively. A p-value of ~~less than 0.05~~ and 95% confidence interval were used to determine statistical significance.<br><br>Results<br><br>The prevalence of safe stool disposal among mothers having under two years of age were 56.3%(95%CI: 54.6%, 58.1%). Mothers with in the age range of 25-34 (AOR= 0.78 (95%CI: 0.62, 0.98)),rich wealth quintile (AOR=0.43 (95%CI:0.33,0.56)), being exposed to media (AOR=1.37 (95%CI: 1.07, 1.76)), increasing age of children (AOR=1.06 (1.05, 1.07)), Being employed (AOR=1.31 (1.11, 1.55)) and Geographic region were significantly associated with safe child disposal practice.<br><br>Conclusion<br><br>The prevalence of safe child stool disposal was low in Gambia. Age of the mother, age of the child, region, wealth index, media exposure and occupational status of the mother were significantly associated with safe child stool disposal. Public health intervention strategies designed to promote safe child stools disposal need to conduct thorough community assessments to identify community-specific facilitators, needs and barriers. Additionally, public health experts and policy makers should take into consideration the geographical and wealth inequalities when designing programs aimed to improve safe child stool disposal practice
**Order of Authors:**	Menen Tsegaw
	Bezawit Mulat

Powered by Editorial Manager® and ProduXion Manager® from Aries Systems Corporation

	Kegnie Shitu
Additional Information:	
Question	Response
**Financial Disclosure**<br><br>Enter a financial disclosure statement that describes the sources of funding for the work included in this submission. Review the submission guidelines for detailed requirements. View published research articles from *PLOS ONE* for specific examples.<br><br>This statement is required for submission and **will appear in the published article** if the submission is accepted. Please make sure it is accurate.<br><br>**Unfunded studies**<br>Enter: *The author(s) received no specific funding for this work.*<br><br>**Funded studies**<br>Enter a statement with the following details:<br>• Initials of the authors who received each award<br>• Grant numbers awarded to each author<br>• The full name of each funder<br>• URL of each funder website<br>• Did the sponsors or funders play any role in the study design, data collection and analysis, decision to publish, or preparation of the manuscript?<br>• **NO** - Include this sentence at the end of your statement: *The funders had no role in study design, data collection and analysis, decision to publish, or preparation of the manuscript.*<br>• **YES** - Specify the role(s) played.<br><br>* typeset	The author(s) received no specific funding for this work
**Competing Interests**<br><br>Use the instructions below to enter a competing interest statement for this submission. On behalf of all authors, disclose any competing interests that could be perceived to bias this work—acknowledging all financial support and any other relevant financial or non-financial competing interests.	The authors have declared that no competing interests exist

Powered by Editorial Manager® and ProduXion Manager® from Aries Systems Corporation

This statement is required for submission and **will appear in the published article** if the submission is accepted. Please make sure it is accurate and that any funding sources listed in your Funding Information later in the submission form are also declared in your Financial Disclosure statement.<br><br>View published research articles from *PLOS ONE* for specific examples.<br><br>**NO authors have competing interests**<br><br>Enter: *The authors have declared that no competing interests exist.*<br><br>**Authors with competing interests**<br><br>Enter competing interest details beginning with this statement:<br><br>*I have read the journal's policy and the authors of this manuscript have the following competing interests: [insert competing interests here]*<br><br>* typeset	
**Ethics Statement**<br><br>Enter an ethics statement for this submission. This statement is required if the study involved:<br><br>• Human participants<br>• Human specimens or tissue<br>• Vertebrate animals or cephalopods<br>• Vertebrate embryos or tissues<br>• Field research<br><br>Write "N/A" if the submission does not require an ethics statement.<br><br>General guidance is provided below. Consult the submission guidelines for detailed instructions. **Make sure that all**	The DHS program conducted the study after getting ethical approval of the Gambia National Ethics Committee. As it is stated in the 2019–20 GDHS report the DHS obtained informed consent from each participant and anonymized the data set during the analysis. Moreover, the data for this study was obtained from the DHS at (http://www.measuredhs.com) after registration and permission had obtained. Since we used secondary data for analysis mentioning the ethical review board and other ethical issues is not applicable. We have obtained permission from the DHS program on reasonable request.

Powered by Editorial Manager® and ProduXion Manager® from Aries Systems Corporation

**information entered here is included in the Methods section of the manuscript.**<br><br>**Format for specific study types**<br><br>**Human Subject Research (involving human participants and/or tissue)**<br>• Give the name of the institutional review board or ethics committee that approved the study<br>• Include the approval number and/or a statement indicating approval of this research<br>• Indicate the form of consent obtained (written/oral) or the reason that consent was not obtained (e.g. the data were analyzed anonymously)<br><br>**Animal Research (involving vertebrate animals, embryos or tissues)**<br>• Provide the name of the Institutional Animal Care and Use Committee (IACUC) or other relevant ethics board that reviewed the study protocol, and indicate whether they approved this research or granted a formal waiver of ethical approval<br>• Include an approval number if one was obtained<br>• If the study involved *non-human primates*, add *additional details* about animal welfare and steps taken to ameliorate suffering<br>• If anesthesia, euthanasia, or any kind of animal sacrifice is part of the study, include briefly which substances and/or methods were applied<br><br>**Field Research**<br><br>Include the following details if this study involves the collection of plant, animal, or other materials from a natural setting:<br>• Field permit number<br>• Name of the institution or relevant body that granted permission	
**Data Availability**<br><br>Authors are required to make all data underlying the findings described fully available, without restriction, and from the time of publication. PLOS allows rare exceptions to address legal and ethical	Yes - all data are fully available without restriction

concerns. See the PLOS Data Policy and FAQ for detailed information.<br><br>A Data Availability Statement describing where the data can be found is required at submission. Your answers to this question constitute the Data Availability Statement and **will be published in the article**, if accepted.<br><br>**Important:** Stating 'data available on request from the author' is not sufficient. If your data are only available upon request, select 'No' for the first question and explain your exceptional situation in the text box.<br><br>Do the authors confirm that all data underlying the findings described in their manuscript are fully available without restriction?	
**Describe where the data may be found in full sentences. If you are copying our sample text, replace any instances of XXX with the appropriate details.**<br><br>• If the data are **held or will be held in a public repository**, include URLs, accession numbers or DOIs. If this information will only be available after acceptance, indicate this by ticking the box below. For example: *All XXX files are available from the XXX database (accession number(s) XXX, XXX.*).<br>• If the data are all contained **within the manuscript and/or Supporting Information files**, enter the following: *All relevant data are within the manuscript and its Supporting Information files.*<br>• If neither of these applies but you are able to provide **details of access elsewhere**, with or without limitations, please do so. For example:<br><br>*Data cannot be shared publicly because of [XXX]. Data are available from the XXX Institutional Data Access / Ethics Committee (contact via XXX) for researchers who meet the criteria for access to confidential data.*	https://dhsprogram.com/data/dataset/Gambia_Standard-DHS_2019.cfm.

The data underlying the results presented in the study are available from (include the name of the third party and contact information or URL).<br>• This text is appropriate if the data are owned by a third party and authors do not have permission to share the data.<br><br>* typeset	
Additional data availability information:	Tick here if the URLs/accession numbers/DOIs will be available only after acceptance of the manuscript for publication so that we can ensure their inclusion before publication.

Powered by Editorial Manager® and ProduXion Manager® from Aries Systems Corporation

Manuscript

Click here to access/download;Manuscript;Safe stool disposal.docx

Safe stool disposal and associated factors among mothers of children under-two age in Gambia: Evidence from Gambia Demographic Health Survey 2019/20

Menen Tsegaw[3], Bezawit Mulat[2] and Kegnie Shitu[1]

Authors' Affiliation

[1]Department of Health Education and\ Behavioral Sciences, Institute of Public Health, College of Medicine and Health Sciences, University of Gondar, Gondar, Ethiopia

[2]Department of Human Physiology, School of Medicine, College of Medicine and Health Sciences, University of Gondar, Gondar, Ethiopia

[3]Department of Public Health, College of Medicine and Health Sciences, Ambo University, Ambo, Ethiopia

***Corresponding author**

Email: menentsegaw@gmail.com

Tel:+251934934533

Abstract

Background: Appropriate disposal of child stool is vital in preventing the spread of faceo-oral diseases. According to WHO/ UNICEF Joint Monitoring Program, Safe child stool disposal includes disposing a stool in to Flush or pour-flush toilet/latrine to (piped sewer system , septic tank , pit latrine), Ventilated improved pit (VIP) latrine and Pit latrine with slab.

Objective: The study aimed to assess safe child stool disposal practice and associated factors among mothers having under two age's children in Gambia

Methods: This study was based on a large community-based cross-sectional survey, conducted from 21 November 2019 to 30 March 2020 in Gambia. The survey employed a two-staged stratified cluster sampling technique to recruit study participants. Descriptive statistics and logistic regression models were used to summarize descriptive data and identify factors associated with safe waste disposal, respectively. A p-value of less than 0.05 and 95% confidence interval were used to determine statistical significance.

Results: The prevalence of safe stool disposal among mothers having under two years of age were 56.3 %(95%CI: 54.6%, 58.1%). Mothers with in the age range of 25-34 (AOR= 0.78 (95%CI: 0.62, 0.98)),rich wealth quintile (AOR=0.43 (95%CI:0.33,0.56)), being exposed to media (AOR=1.37 (95%CI: 1.07, 1.76)), increasing age of children (AOR=1.06 (1.05, 1.07)), Being employed (AOR=1.31 (1.11, 1.55)) and Geographic region were significantly associated with safe child disposal practice.

Conclusion: The prevalence of safe child stool disposal was low in Gambia. Age of the mother, age of the child, region, wealth index, media exposure and occupational status of the mother

were significantly associated with safe child stool disposal. Public health intervention strategies designed to promote safe child stools disposal need to conduct thorough community assessments to identify community-specific facilitators, needs and barriers. Additionally, public health experts and policy makers should take into consideration the geographical and wealth inequalities when designing programs aimed to improve safe child stool disposal practice

Key words: safe stool disposal, Gambia

Introduction

According to WHO/ UNICEF Joint Monitoring Program, Safe child stool disposal includes Flush or pour-flush toilet/latrine to(piped sewer system , septic tank , pit latrine), Ventilated improved pit (VIP) latrine and Pit latrine with slab whereas unsafe stool disposal includes pit latrines without a slab or platform, hanging latrines and bucket latrines (1)(2). Appropriate disposal of child stool is vital in preventing the spread of faceo-oral diseases(3)(4)(5). Sustainable development Goal (SDG) 6 aims to "achieve access to adequate and equitable sanitation and hygiene for all and end open defecation, paying special attention to the needs of women and girls and those in vulnerable(6.2) by 2030 "(6). Achieving the widespread scope of SDG by 2030 needs quadrupling current rates of advance in securely overseen drinking water administrations, sanitation administrations and basic hygiene services especially in least developing countries (7). Elimination of open defecation is one of the core priority solutions to reduce global disparities in Water, Sanitation, and Hygiene (WASH) (8).

Different studies revealed that unsafe child stool disposal was one of the major factors that causes enteric infections including Diarrheal diseases (3)(9)(10,11)(12). Diarrhea is one of the most common causes of under-five mortality rate (13)(14). There is a common misunderstanding that child faeces are less infectious than adult (15)(16). Besides the Water and Sanitation Project implemented by the Department of Water Resources, to introduce hygienic means of excreta disposal in the entire country, there has not been much-coordinated policy response to basic sanitation issues in the country (17). Different studies have been done on safe child stool disposal and associated factors among children but there is no study done in Gambia. Among the studies that investigated child feces disposal, most found that the age of the child, maternal age, maternal education, household wealth index, number of children, number of children,sex of the child,

availability of toilet facility, improved drinking water, number of living under-five children and history of diarrhea (18)(19)(20). According to the Gambia Demographic Health Survey (GDHS) 2019/20, safe child stool disposal among children under the age of 2 years has been decreased from 82% in 2013 to 56% in 2019-20. Although the magnitude of safe child stool disposal is known the possible factors affecting safe child stool disposal have not yet been well studied. Therefore, the current study is aimed to assess factors that are significantly associated with safe child stool disposal practice in Gambia using the GDHS 2019-20.

Methods

Study design and setting

This study was based on a large community-based survey, Gambia Demographic Health Survey (GDHS), conducted from 21 November 2019 to 30 March 2020 in Gambia. Gambia is located on the West African coast. It is bordered on the North, South and East by the Republic of Senegal and on the West by the Atlantic Ocean. The country has a tropical climate characterized by two seasons: rainy season (June – October) and dry season (November-May)(21). The survey employed a stratified two-stage cluster sampling. In the first stage, EAs were selected with a probability proportional to their size within each sampling stratum. In the second stage, the households were systematically sampled. A total of 3,011-weighted sample of mothers who have child/children under the age of two years were included in the study.

Variables of the study

Dependent variable

Safe child stool disposal was the outcome variable of this study which was obtained after categorizing child stool disposal in to two as safe **(**Flush or pour-flush toilet/latrine to(piped sewer system , septic tank , pit latrine), Ventilated improved pit (VIP) latrine and Pit latrine with slab **) and unsafe(**pit latrines without a slab or platform, hanging latrines and bucket latrines**)**

Independent variables

Age of the mother, religion, region, educational level, occupation, residence, wealth index, media exposure (yes, no), age of the child, toilet facility(improved, unimproved), sex of the child, birth order, visited a health facility in the last 12 months (yes, no), history of diarrhea in the last 2 weeks preceding the survey (yes, no), source of drinking water(improved, unimproved) and the number of living children were included in the study. Those variables were identified after reviewing different literature (5,15,18,19,22).

Data analysis

The women's individual sample weightings were used in the estimation to provide nationally representative results. Descriptive studies like frequency count and proportion for categorical data were used to summarize descriptive data. Bivariable logistic regression was used to select candidate variables for multivariable logistic regression. In the Bivariable logistic regression, a p-value of less than 0.2 was used as a cut of point to select variables for the multi-variable analysis entry. Multivariable logistic regression was used to identify independent predictors of safe child stool disposal in Gambia controlling confounders. A 95% confidence interval (CI) and p-value<0.05 were used to determine the statistical significance. Stata version 14.0 was used for statistical analysis.

Results

The mean age of the mothers was 28.35 with a standard deviation of 6.65 and range of 15-49 years. Regarding religion, 2,952 (98.02%) of mothers were Muslims. About 1,946 (64.61%) of the mothers were from urban, 1202 (39.92%) were from Brikama region, 1,337 (44.39%) of mothers have no education,1177 (39.08) of mothers were employed and 1314 (43.63%) of mothers were from the poor families. Regarding media exposure 2,759 (91.62%) of mothers were exposed to media. Over half 1851 (61.46%) of households used improved toilet facilities. The majority, 2677 (88.90%) of the households used improved source of drinking water. Most, 2768 (91.91%) of the mothers have visited health facilities in the last 12 months. Regarding diarrhea prevalence, 801 (26.58%) of children experienced diarrhea in the last two weeks preceding the survey (Table1).

Table1 Socio-demographic characteristics by waste disposal status among mothers/caregivers of children with the age < 2 years in Gambia, 2019/20 (n=3011)

Variables	Child waste disposal practice				Total		p-value
	Unsafe		Safe				
	Frequency	Percent	frequency	Percent	Frequency	Percent	
Age of the mother							
15-24	367	43.82	471	56.18	838	27.84	0.08

25-34	700	44.50	874	55.50	1574	52.28	
>34	246	41.07	353	58.93	599	19.89	
Region		-		-			
Banjul	17	66.91	8	33.09	26	0.85	
Kaniking	348	68.68	159	31.32	507	16.85	
Brikama	559	46.47	643	53.53	1202	39.92	<0.001
Mansakonko	23	18.14	106	81.86	129	4.29	
Kerewan	153	41.20	219	58.80	372	12.34	
Kuntaur	78	41.14	111	58.86	189	6.27	
Janjanbureh	40	21.02	152	78.98	193	6.40	
Basse	95	24.06	299	75.94	394	13.08	
Mothers educational level		-		-			
No education	514	38.48	822	61.52	1337	44.39	<0.001
Primary	225	39.30	348	60.70	574	19.05	
Secondary	502	51.44	474	48.56	976	32.42	
Higher	72	57.51	53	42.49	125	4.14	
Occupation							
Employed	612	51.98	565	48.02	1177	39.08	<0.001
Unemployed	702	38.27	1132	61.73	1834	60.92	
Religion							
Islam	1284	43.51	1668	56.49	2952	98.02	0.15
Christian	30	49.64	30	50.36	60	1.98	

Residence							
Urban	996	51.22	949	48.78	1946	64.61	<0.001
Rural	317	29.78	748	70.22	1066	35.39	
Wealth index							
Poor	427	32.25	887	52.24	1314	43.63	<0.001
Middle	199	15.14	430	25.34	629	20.89	
Rich	688	52.34	380	22.46	1068	35.48	
Media exposure		-		-			
No	91	6.93	161	9.49	252	91.62	<0.223
Yes	1223	93.07	1536	90.51	2759	91.62	
Age of the child		-		-			
0-5 months	502	56.05	394	43.95	897	29.77	<0.001
6 to 11 months	348	47.53	384	52.47	732	24.31	
12 to 17 months	289	36.66	499	63.34	787	26.14	
18 to 23 months	175	29.34	421	70.66	596	19.78	
Sex of the child		-		-			
Male	663	42.60	893	57.40	1556	51.66	0.69
Female	651	44.73	804	55.27	1456	48.34	
Toilet facility		-		-			
Unimproved	388	33.41	773	66.59	1161	38.54	<0.001
Improved	926	50.03	925	49.97	1851	61.46	
Source of drinking water		-		-			

Unimproved	136	40.58	199	59.42	334	11.10	0.84
Improved	1178	44.01	1499	55.99	2677	88.90	
History of Diarrhea		-		-			
No	988	44.67	1223	55.33	2211	73.42	0.27
Yes	326	40.74	474	59.26	801	26.58	
Birth order		-		-			
First	307	49.13	318	50.87	625	20.75	<0.001
Second	260	47.18	291	52.82	552	18.32	
Third	241	46.17	281	53.83	523	17.35	
Fourth and above	505	38.50	807	61.50	1312	43.58	
Visited health facility		-		-			
No	115	4.17	128	4.63	2768	91.92	0.08
Yes	1198	43.30	1569	56.70	2768	91.91	
Number of living children		-		-			
1 child	318	48.02	344	51.98	662	21.97	<0.001
2 children	287	49.07	297	50.93	584	19.40	
3 children	249	45.83	295	54.17	544	18.05	
4 and above	460	37.67	762	62.33	1222	40.58	

Prevalence of safe Children’s stools disposal

The prevalence of safe stool disposal among mothers whose child aged under two years was 56.3% (95%CI: 54.5, 58.1%) (Table 2).

Table2; shows the percentage of youngest children's stools disposal among mothers whose child aged under two years using GDHS 2019/20 (n=3011).

variables	Weighted frequency	Weighted percent (95% CI)
Type of toilet facility		
Used toilet/latrine	1697	56.3(54.5, 58.1)
Put/rinsed into drain/ditch	190	6.3 (5.4,7.2)
Throw into garbage	1097	36.4 (34.7,38.1)
Left in the open/not disposed of	22	0.8 (0.4,1.1)
Other	5	0.2 (0.0,0.4)
Child waste disposal		
Safe	1697	56.3 (54.5, 58.1)
Unsafe	1314	43.7 (41.8, 45.4)

Factors associated with safe children's stool disposal

In the Bivariable logistic regression age of the mother, religion, region, educational level, occupation, residence, wealth index, media exposure, age of the child, toilet facility, birth order, visited health facility in the last 12 months, and the number of living children, sex of the child, diarrhea history in the last 12months and source of drinking water were independently fitted to the outcome variable. However, only age of the mother, religion, region, educational level, occupation, residence, wealth index, media exposure, age of the child, toilet facility, birth order,

visited health facility in the last 12 months, and the number of living children exhibited an association at p-value less than 0.2 and passed to be entered in to the multivariable logistic regression.

In the multivariable logistic regression, age of the mothers, occupational status of the mother, household wealth index, region, age of the child and media exposure were significantly associated with safe child stool disposal at p-value of less than 0.05.

The odds of safe stool disposal was 22% (AOR=0.78 (95%CI: 0.62, 0.98)) lower among mothers aged 25-34 as compared with mothers aged 15-24 year. Region of mothers were also significantly associated with safe stool disposal. The odds of disposing stool were 2.33 (AOR=2.33 (95%CI:1.55,3.50)),5.98(AOR=5.98(95%CI:3.60,9.94)),1.90(AOR=1.90(95%CI:122,2.97)),1.64 (AOR=1.64(1.03,2.61),4.48(AOR=4.42(95%CI:2.77, 7.25)),5.01(AOR=4.79(95%CI:3.23,7.78)) times higher among respondents in Brikama, mansakonko, kerewan , kuntaur, janjanbureh, and Basse respectively as compared to Banjul.

The odds of safe stool disposal were 56% (AOR=0.43,95%CI:0.44,0.88) lower among rich households as compared to those with poor wealth quantiles. The odds of safe stool disposal was 1.31 (AOR=1.31 (95%CI: 1.11, 1.55)) times higher among employed women as compared with unemployed mothers .Age of the child was significantly associated with safe stool disposal. As the age of the child increases the odds of safe stool disposal increases by 6% (AOR=1.06 (95%CI: 1.05, 1.07).

Media exposure were significantly associated with safe stool disposal. The odds of safe stool disposal were 1.37 (AOR=1.37 (95%CI: 1.07, 1.76)) times higher among mothers who were exposed to at least one form of media as compared with their counterparts (Table 3).

Table3: Factors associated with safe stool disposal among mothers whose children aged under two years using GDHS, 2019/20 (n=3011)

Variables	COR(95%CI)	P-value	AOR(95%CI)	p-value
Age of the mother				
15-24	1		1	
25-34	0.78 (0.79,1.09)	0.42	0.78 (0.62, 0.98)*	0.033
>34	1.16 (0.94,1.42)	0.17	0.75 (0.53, 1.04)	0.088
Region				
Banjul	1		1	
Kaniking	0.93 (0.61,1.41)	0.73	1.04 (0.67, 1.62)	0.869
Brikama	2.44 (1.66,3.570	<0.001	2.33 (1.55, 3.50)*	<0.001
Mansakonko	9.10 (5.60,14.26)	<0.001	5..98(3.60, 9.94)*	<0.001
Kerewan	2.86 (1.93,4.22)	<0.001	1.90 (1.22, 2.97)*	0.005
Kuntaur	2.70 (1.83,3.98)	<0.001	1.64 (1.03, 2.61)*	0.037
Janjanbureh	6.60 (4.36,10.01)	<0.001	4.48 (2.77, 7.25)*	<0.001
Basse	6.75 (4.58,9.94)	<0.001	5.01 (3.23, 7.78)*	<0.001
Mothers educational level				
No education	1		1	
Primary	1.04 (0.86, 1.31)	0.71	1.09 (0.89, 1.37)	0.389
Secondary	0.61 (0.52, 0.72)	<0.001	0.97 (0.79, 1.18)	0.747
Higher	0.44 (0.31, 0.61)	<0.001	1.06 (0.65, 1.75)	0.809

Occupation				
Unemployed	1		1	
Employed	1.60 (1.38, 1.85)	<0.001	1.31 (1.11,1.55)*	0.001
Religion				
Islam	1		1	
Christian	0.63 (0.34, 1.18)	0.15	1.09 (0.54, 2.20)	0.801
Residence				
Urban	1			
Rural	2.27 (1.96,2.62)	<0.001	1.16 (0.90, 1.48)	0.249
Wealth index				
Poor	1		1	
Middle	1.00 (0.83, 1.22)	0.975	1.16(0.92, 1.48)	0.208
Rich	0.29 (0.24, 0.34)	<0.001	0.43(0.33, 0.56)*	<0.001
Media exposure				
No	1			
Yes	0.87 (0.69 , 1.08)	0.223	1.37 (1.07, 1.76)*	0.014
Age of the child	1.09 (1.06, 1.13)	0.001	1.06 (1.05, 1.07)	<0.001
Sex of the child				
Male	1			
Female	0.97 (0.84, 1.12)	0.69	-	
Toilet facility				
Unimproved	1			
improved	0.61 (0.53,0.70)	<0.001	1.14 (0.95, 1.36)	0.150

Source of drinking water				
Unimproved	1			
improved	0.98 (0.78,1.22)	0.84	-	
Diarrhea in the last two weeks				
No	1			
Yes	1.09 (0.93, 1.28)	0.27	-	
Birth order				
First	1			
Second	1.08 (0.86,1.36)	0.49	1.06 (0.81, 1.38)	0.674
Third	1.08 (0.86,1.36)	0.49	1.07 (0.78, 1.47)	0.680
Fourth and above	1.48 (1.23,1.78)	<0.001	1.39 (0.93, 2.10)	0.106
Visited health facility				
No	1			
Yes	1.25 (0.97,1.60)	0.08	1.08 (0.81, 1.42)	0.600
Number of children born	1.09 (1.06, 1.13)	<0.001	1.01 (0.93, 1.11)	0.841

Note: (-): Not applicable for AOR, (*): statistically significant at p-value<0.05

Discussions

The study aimed to assess safe child stool disposal and associated factors among mothers having a child under the age of two years. The prevalence of safe stool disposal among mothers whose child was aged below two years of age was 56.3 %(95%CI: 54.5%, 58.1%)). The finding of the study is higher than ~~studies done~~ in Eswatini , Ethiopia, Bangladesh, India, and Orissa

(3,15,20,23,24). On the other hand~~s~~, the finding of the current study is lower than the study conducted in Malawi, Cambodia and Sub-Saharan Africa (19,25,26). The possible reason for the discrepancies might be differences in sample size, sampling technique, method of analysis and differences in population characteristics. The study conducted in Sub-Saharan Africa used large sample size and it incorporates individual and contextual factors of different countries that might affect safe stool disposal practice. ~~With-in and across-country~~ contextual variations and commonalities might affect safe disposal of children's faeces (19,27).

Region of the mother is associated with safe stool disposal. Mothers from Brikama, mansakonko, kerewan, kuntaur, janjanbureh, Basse were more likely to disposed stool safely as compared with those from Banjul region of Gambia. The possible reason might be different regions of a country may have different access to health care service, infrastructure and different level of health literacy.

Safe stool disposal was significantly associated with the age of the mother. Women in the age group of 25-34 years were less likely to dispose child's stool safely as compared with women in the age group of f 15-24 years. The finding is consistent with the studies done in Nigeria (28). This finding is inconsistent with studies done in Sub-Saharan Africa(19). The possible reason for the discrepancy might be that the study done in sub-Saharan Africa used mothers having under-five children in 15 sub-Saharan countries, but our study used only mothers of children under the age of 2 years in Gambia. The other possible reason might be time variation in which our study used only 2019/20 GDHS data but the study in sub-Saharan Africa used DHS data 2015-2018 in 15 sub-Saharan countries.

Safe stool disposal was associated with the age of children. The odds of safe stool disposal increases with increasing child age. This is finding is consistent with the studies done in Malawi,

Ethiopia, Eswatini and Ghana (15,18,25,29) .The possible reason might be that as children get older especially starting from 6 months as they start complementary feeding their stool becomes offensive which increases the likelihood of safe stool disposal in older children as compared to younger children less than 6 month whose stool is not offensive making it favorable to defecate on clothes/diapers.

Those from rich households were less likely to dispose of stool as compared with those from the poor wealth quantiles. Although many studies have revealed that, the highest wealth quantile increases the likelihood of safe waste disposal (16,20,22,30). The findings of our study are in contrary to those findings. Even though having the highest wealth is important to have easy access to water, sanitation, and hygiene services, it is not a guarantee for safe stool disposal. The highest wealth quantile is necessary but not sufficient to dispose of stools safely. Other factors like knowledge and perception might affect safe stool disposal behavior.

The occupational status of the mothers was significantly associated with safe stool disposal. Mothers who were employed were more likely to dispose of stool safely as compared with unemployed mothers. The finding is inconsistent with a cross-sectional study done in Tigray region of Ethiopia (31). The possible reason for the discrepancy might be the smaller sample size included in Tigray and the large representative sample used in our study. The other possible reason might be employed mothers have their source of income and they can hire caregivers as well as they can easily access sanitary materials. Therefore, employed mothers would be more likely to dispose of stool safely than mothers who are not employed.

Media exposure was associated with safe child stool disposal. Mothers who were exposed to a media at least once a week were more likely to dispose of stool safely as compared with those who had not exposed. The finding of the study is consistent with what has been reported by a study in ~~in~~ sub-Saharan Africa (19). Mothers who ~~were~~ exposed to media might ~~get~~ important health information about child waste disposal and its impact on the health of the child as well as the community as a whole, so they might ~~have~~ good knowledge about safe child waste disposal and develop a positive attitude towards the importance of safe child disposal behavior ~~than~~ those mothers ~~who do~~ not exposed. Media ~~has~~ a great impact on behavior change by providing health information to shape the attitude of the community and to promote healthy behavior. Especially Audio-visual education ~~has~~ a great impact on increasing safe child stool practice (19).

~~Strength~~

The application of sample weighting to overcome non-proportional sample allocation during the survey. Another strength of the current study is the use of a large sample size that can help to increase the statistical power and validity of the study. Utilization of large sample size and nationally representativeness of DHS data helps to generalize to the population of Gambia.

~~Limitations~~

The main limitation of the study is that since it is a cross-sectional study the temporal relationship between the outcome and independent variables could not be established. The DHS data lacks some variables like knowledge about child stool disposal that may affect their disposal practice. There might also be social desirability bias and recall bias.

Conclusion

The prevalence of safe child stool disposal was low in Gambia. Age of the mother, age of the child, region, wealth index, media exposure and occupational status of the mother were significantly associated with safe child stool disposal. Public health intervention strategies designed to promote safe child stools disposal need to conduct thorough community assessments to identify community-specific facilitators, needs and barriers. Additionally, public health experts and policy makers should take into consideration the geographical and wealth inequalities when designing programs aimed to improve safe child stool disposal practice

Declaration

Acronyms: COR (Crude Odds Ratio)), AOR (Adjusted Odds Ratio) CI: Confidence interval: GDHS: Gambia Demographic Health Survey

Ethics approval and consent to participate

The DHS program conducted the study after getting ethical approval of the Gambia National Ethics Committee. As it is stated in the 2019–20 GDHS report the DHS obtained informed consent from each participant and anonymized the data set during the analysis. Moreover, the data for this study was obtained from the DHS at (http://www.measuredhs.com) after registration and permission had obtained. Since we used secondary data for analysis mentioning the ethical review board and other ethical issues is not applicable. We have obtained permission from the DHS program on reasonable request.

Consent for publication

Not applicable

Availability of data and material

The datasets used and/or analyzed during the current study are publicly available and can access it from https://dhsprogram.com/data/dataset/Gambia_Standard-DHS_2019.cfm.

Competing interest

The authors declare that they have no competing interests.

Funding

None

Author's contribution

MT, KS, and BM contributed to the study conceptualization and provided critical editorial input to the interpretation of the data. MT conduct the formal analysis and write the draft manuscript. MT, KS, and BM reviewed the drafted manuscript. All authors read and approved the final manuscript.

Acknowledgement

We would like to express our gratitude to Demographic Health survey (DHS) program for giving us 2019/20 GDHS data. We also would like to thank study participants.

References

1. Soboksa NE. Associations Between Improved Water Supply and Sanitation Usage and Childhood Diarrhea in Ethiopia: An Analysis of the 2016 Demographic and Health Survey. Environ Health Insights. 2021;15.

2. WHO/UNICEF. Progress on drinking-water and sanitation [Internet]. Joint Monitoring Programme for water supply and sanitation …. New York and WHO, Geneva; 2008. Available from:

https://www.who.int/water_sanitation_health/monitoring/jmp_report_7_10_lores.pdf

3. Bawankule R, Singh A, Kumar K, Pedgaonkar S. Disposal of children's stools and its association with childhood diarrhea in India. BMC Public Health [Internet]. 2017;17(1):1–9. Available from: http://dx.doi.org/10.1186/s12889-016-3948-2

4. George CM, Oldja L, Perin J, Sack RB, Biswas S, Ahmed S, et al. Unsafe Child Feces Disposal is Associated with Environmental Enteropathy and Impaired Growth. J Pediatr [Internet]. 2016;176:43–9. Available from: http://dx.doi.org/10.1016/j.jpeds.2016.05.035

5. Azage M, Haile D. Factors associated with safe child feces disposal practices in Ethiopia: Evidence from demographic and health survey. Arch Public Heal. 2015;73(1):198–204.

6. Bárcena A, Cimoli M, García-Buchaca R, Yáñez LF, Pérez R. The 2030 Agenda and the Sustainable Development Goals An opportunity for Latin America and the Caribbean Thank you for your interest in this ECLAC publication. 2018.

7. UNICEF/WHO. Progress on Household Drinking Water , [Internet]. 2021. Available from: https://washdata.org/sites/default/files/2021-07/jmp-2021-wash-households.pdf

8. UNICEF, World Bank. Child Feces Disposal in Mozambique. 2105;(December):1–4. Available from: http://documents.worldbank.org/curated/en/892471467992479942/pdf/96434-BRI-CHILD-FECES-Box391444B-PUBLIC-WSP-Mozambique-CFD-Profile.pdf

9. Edwin P, Azage M. Geographical Variations and Factors Associated with Childhood Diarrhea in Tanzania: A National Population Based Survey 2015-16. Ethiop J Health Sci. 2019;29(4):513–24.

10. Mihrete TS, Alemie GA, Teferra AS, Access O, Cheng SX, Zhao Y, et al. Prevalence of diarrhea and associated risk factors in children under five years of age in Northern Nigeria: A secondary data. Nord J African Stud [Internet]. 2017;16(1):64–74. Available from: http://www.diva-portal.org/smash/get/diva2:1106657/FULLTEXT01.pdf%0Ahttp://www.njas.helsinki.fi/pdf-files/vol16num1/asakitipki_alex.pdf%0Ahttp://dx.doi.org/10.1186/s41043-016-0052-2%0Ahttp://dx.doi.org/10.1186/s40249-016-0157-2%0ABMC Medicine%0Ahttp://www

11. Alemayehu K, Oljira L, Demena M, Birhanu A, Workineh D. Prevalence and Determinants of Diarrheal Diseases among Under-Five Children in Horo Guduru Wollega Zone, Oromia Region, Western Ethiopia: A Community-Based Cross-Sectional Study. Can J Infect Dis Med Microbiol. 2021;2021.

12. Natnael T, Lingerew M, Adane M. Prevalence of acute diarrhea and associated factors among children under five in semi-urban areas of northeastern Ethiopia. BMC Pediatr. 2021;21(1):1–15.

13. Hossain MJ, Saha D, Antonio M, Nasrin D, Blackwelder WC, Ikumapayi UN, et al. Cryptosporidium infection in rural gambian children: Epidemiology and risk factors. PLoS Negl Trop Dis. 2019;13(7):1–16.

14. Laham N Al, Elyazji M, Al-Haddad R, Ridwan F, Anteneh ZA, Andargie K, et al. Factors related to the occurrence of diarrheal disease among under-five children in Lalitpur district of Nepal. BMC Public Health [Internet]. 2014;11(1):1–6. Available from: https://doi.org/10.4236/oalib.1100283%0Ahttp://www.embase.com/search/results?subaction=viewrecord&from=export&id=L358361323%0Ahttp://sfx.eu.novartis.net:9004/sfx_lo

cal?&sid=EMBASE&issn=16060997&id=doi:&atitle=Prevalence+of+intestinal+parasitic+and+bacteri

15. Simelane MS, Chemhaka GB, Maphosa T, Zwane E. Unsafe disposal of faeces and its correlates among children under three years in Eswatini. SAJCH South African J Child Heal. 2020;14(4):217–23.

16. Sahiledengle B. Trend and predictors of unsafe child stool disposal in Ethiopia (2000-2016): pooled evidence from demographic and health survey. 2019;1–20.

17. REPUBLIC OF THE GAMBIA MINISTRY OF HEALTH AND SOCIAL WELFARE THE GAMBIA NATIONAL STRATEGY Banjul March 2011. 2016;(March 2011).

18. Seidu AA. Are children's stools in Ghana disposed of safely? Evidence from the 2014 Ghana demographic and health survey. BMC Public Health. 2021;21(1):1–10.

19. Seidu AA, Ahinkorah BO, Kissah-Korsah K, Agbaglo E, Dadzie LK, Ameyaw EK, et al. A multilevel analysis of individual and contextual factors associated with the practice of safe disposal of children's faeces in sub-Saharan Africa. PLoS One [Internet]. 2021;16(8 August):1–17. Available from: http://dx.doi.org/10.1371/journal.pone.0254774

20. Sahiledengle B. Unsafe child feces disposal status in Ethiopia: What factors matter? Analysis of pooled data from four demographic and health surveys. BMC Public Health. 2020;20(1):1–12.

21. Hotel CP. The Gambia country profile - Overview. 2015;1–6. Available from: http://www.bbc.com/news/world-africa-13376517

22. Sahiledengle B. Prevalence and associated factors of safe and improved infant and young

children stool disposal in Ethiopia: Evidence from demographic and health survey. BMC Public Health. 2019;19(1):1–13.

23. Islam M, Ercumen A, Ashraf S, Rahman M, Shoab AK, Luby SP, et al. Unsafe disposal of feces of children <3 years among households with latrine access in rural Bangladesh: Association with household characteristics, fly presence and child diarrhea. PLoS One. 2018;13(4):1–13.

24. Majorin F, Freeman MC, Barnard S, Routray P, Boisson S, Clasen T. Child feces disposal practices in rural Orissa: A cross sectional study. PLoS One. 2014;9(2):1–7.

25. Nkoka O. Correlates of appropriate disposal of children's stools in Malawi: A multilevel analysis. BMC Public Health. 2020;20(1):1–10.

26. Vong P, Banchonhattakit P, Sim S, Pall C, Dewey RS. Unhygienic stool-disposal practices among mothers of children under five in Cambodia: Evidence from a demographic and health survey. PLoS One [Internet]. 2021;16(7 July):1–13. Available from: http://dx.doi.org/10.1371/journal.pone.0249006

27. Demissie GD, Yeshaw Y, Aleminew W, Akalu Y. Diarrhea and associated factors among under five children in sub-Saharan Africa: Evidence from demographic and health surveys of 34 sub-Saharan countries. PLoS One [Internet]. 2021;16(9 September):1–13. Available from: http://dx.doi.org/10.1371/journal.pone.0257522

28. Aliyu A, Dahiru T. Factors associated with safe disposal practices of child's faeces in Nigeria: Evidence from 2013 Nigeria demographic and health survey. Niger Med J [Internet]. 2019 Jul 1;60(4):198–204. Available from: https://www.nigeriamedj.com/article.asp?issn=0300-1652

29. Sahiledengle B, Teferu Z, Tekalegn Y, Awoke T, Zenbaba D, Bekele K, et al. Geographical variation and factors associated with unsafe child stool disposal in Ethiopia: A spatial and multilevel analysis. PLoS One [Internet]. 2021;16(4 April):1–19. Available from: http://dx.doi.org/10.1371/journal.pone.0250814

30. Azage M, Haile D. Factors associated with safe child feces disposal practices in Ethiopia: Evidence from demographic and health survey. Arch Public Heal. 2015;73(1):1–9.

31. Ayele Y, Yemane D, Redae G, Mekibib E. Child feces disposal practice and associated factors: A dilemma in Tigray, northern Ethiopia. J Water Sanit Hyg Dev. 2018;8(1):62–70.
